# Extending the Coverage of IEEE 802.15.4z HRP UWB Ranging

**DOI:** 10.3390/s25103058

**Published:** 2025-05-12

**Authors:** Sumin Han, Byung-Jun Jang

**Affiliations:** Department of Electrical Engineering, Kookmin University, Seoul 02707, Republic of Korea; hsm2005x@kookmin.ac.kr

**Keywords:** UWB, HRP UWB, positioning, ranging, IEEE 802.15.4z, link budget, IoT

## Abstract

Currently, the ultra-wideband (UWB) technology commercialized in smartphones and smart keys is a high-rate pulse repetition frequency (HRP) UWB of the IEEE 802.15.4z standard, which aims to accurately determine the distance between UWB devices located within tens of meters using two-way ranging (TWR). However, in order for UWB ranging technology to be spread to various location-based services or positioning services, it must be able to measure the distance between UWB devices that are hundreds of meters apart. Fortunately, UWB technology can freely change physical layer parameters, as long as they comply with the UWB local regulations worldwide. Therefore, in this study, we analyzed the method of configuring the packet structure, length, and transmission power from the link budget perspective to enable longer UWB ranging of hundreds of meters within UWB local regulations. As a result of the analysis, we theoretically confirmed that UWB ranging is possible even at hundreds of meters by selecting the optimal physical layer parameters. In addition, the experimental results using the Qorvo DW3000 module were confirmed to be consistent with the results analyzed in this study. The results of this study can be used as basic data for the introduction of wide-area UWB technology and services in the future.

## 1. Introduction

Ultra-wideband (UWB) technology has gained attention in various fields due to its potential for precise distance measurement and ranging. In particular, high-rate pulse repetition frequency (HRP) UWB, defined in the IEEE 802.15.4z standard, has recently become prominent for enabling centimeter-level ranging accuracy between UWB devices [1,2]. This UWB technology first appeared as short-range wireless personal area network (WPAN) technology in the early 2000s. It was standardized by the IEEE 802.15.4 committee, a WPAN standardization organization, and was announced as the IEEE 802.15.4a standard in 2007. However, UWB technology has not been widely adopted as WPAN communication technology due to competing technologies such as Wi-Fi and Bluetooth [3,4]. However, with the increasing demand for location-based services in the IoT, smart factories, and other applications, UWB technology has been re-evaluated for its advantage of being able to measure the distance between UWB devices in addition to providing wireless communication. Since Apple’s iPhone 11 in 2019, which launched a service that informs users of the locations of nearby UWB devices, most smartphones released by Samsung, Apple, and Google on the market today have integrated HRP UWB technology. In addition, automobile companies such as Hyundai Motors are also introducing UWB smart keys [5,6]. Following this trend, HRP UWB technology has continued to improve its performance with the announcement of the IEEE 802.15.4z standard in 2020, and the IEEE 802.15.4ab standardization work is currently in progress [5,6,7,8,9,10].

Since HRP UWB ranging was originally introduced as a short-range precision ranging solution and not a long-range communication solution, there has been no meaningful attempt to expand the ranging coverage. Moreover, since HRP UWB ranging has been mainly applied to smartphones, its function has been limited to measuring the exact distance between nearby UWB devices after pairing via Bluetooth. Most smartphones first recognize UWB devices through Bluetooth pairing before performing UWB ranging to reduce power consumption in UWB ranging operation. As a result, HRP UWB ranging was constrained to several tens of meters, equivalent to the Bluetooth communication range. This trend is also true for HRP UWB applications other than smartphones. Most of the existing studies on IEEE 802.15.4z HRP UWB investigated by the authors have focused on improving the ranging accuracy, ensuring communication robustness, and mitigating interference with Wi-Fi 6E [11,12,13,14,15,16,17,18,19,20,21]. Table 1 summarizes the operation ranges reported in prior studies, with most commercially available modules (e.g., Qorvo’s DWM1000, DW3000, and DW3120; NXP’s SR150 and SR040; and Spark’s SR1010 and SR1020) being limited to approximately 50 or 100 m.

Nevertheless, for IEEE 802.15.4z UWB technology to be widely used beyond smartphones and smart keys, it must be able to measure the distance between UWB devices that are hundreds of meters apart. If a longer operating range is possible beyond the current operating range limit of tens of meters, then HRP UWB communication technology can be widely used in various fields such as logistics, smart factories, and smart cities. In an effort to achieve this, alternative approaches such as hybrid localization schemes combining UWB ranging with inertial measurements [28] or control-based estimation methods [29] have been proposed. These methods have demonstrated effectiveness in mitigating packet loss and maintaining tracking continuity under degraded link conditions. Although hybrid localization schemes and sensor fusion techniques have shown promise in mitigating ranging failures under degraded conditions, they inherently introduce additional complexity, cost, and energy consumption to the system. In many practical deployments, such as low-power IoT devices, logistics networks, or cost-sensitive industrial applications, it is highly desirable to maximize UWB ranging capabilities using the existing hardware alone, without reliance on auxiliary sensors or external data fusion.

However, extending the operating range purely through physical layer parameter optimization is particularly challenging due to the inherent link performance imbalance between the synchronization and data transmission portions of the UWB packet. This study specifically addresses these challenges by performing a detailed link budget analysis and experimentally validating a PHY-only optimization strategy, demonstrating that long-range UWB operation can be achieved while maintaining regulatory compliance and minimal system complexity.

In this study, we focus on extending the fundamental ranging coverage of IEEE 802.15.4z HRP UWB purely through physical layer parameter optimizations, without relying on auxiliary sensors or external estimation techniques. We propose a method to extend the ranging capability of IEEE 802.15.4z HRP UWB systems to hundreds of meters. This is achieved through a detailed link budget analysis considering the packet structure, packet length, and transmission power within worldwide UWB local regulations. Our results show that by optimizing the physical and RF parameters based on a detailed link budget analysis that accounts for the inherent link performance imbalance between the SYNC (preamble) and the data portion (DP), UWB systems can achieve significantly extended ranging without violating power emission limits. This improvement is not merely a result of increasing the output power but rather addressing the link performance difference. By reducing the link differences between the SYNC and the DP, the overall packet reception performance is improved. Thus, the extended ranging capability is quantitatively supported by a more balanced and favorable link budget distribution across the full packet structure. We also experimentally verify the proposed method using Qorvo DW3000, and the experimental results are consistent with the theoretical analysis. Our experimental results show that by tuning these parameters while respecting the local regulation power constraints, a longer UWB operating range can be achieved.

The composition of this paper is as follows. First, in Section 2, we explain the characteristics of HRP UWB technology based on UWB local regulations. Then, in Section 3, we analyze the link performance of IEEE 802.15.4z HRP UWB communication and analyze the parameters for long-range measurement of HRP UWB technology. In Section 4, we describe the experimental method and experimental results to verify these analysis results, and finally, in Section 5, we conclude the paper.

## 2. UWB Local Regulations and UWB Pulse Characteristics

**Figure 1:**
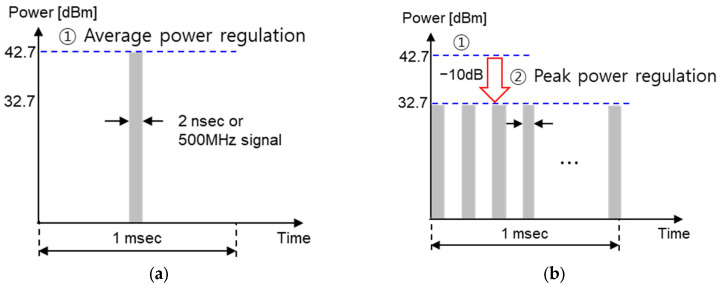
Local regulation for output power of UWB transmitter (**a**) in the case of one pulse and (**b**) in the case of multiple pulses.

UWB means using ultra-wideband technology, and the local regulations in Table 2 must be applied worldwide. As shown in Table 2, UWB involves frequency-related regulations, such as the frequency band or frequency bandwidth used, and output power, such as average power and peak power. Among the local regulations in Table 2, the output power regulations are applied globally; that is, the output power of the UWB transmitter must satisfy both ① the average power spectral density (PSD) of −41.3 dBm/MHz at 1 ms and ② the peak PSD of 0 dBm/50 MHz. This is explained in Figure 1. Assuming that one pulse has a bandwidth of 500 MHz, the pulse width is approximately 2 ns. The peak power of one 2-ns pulse must be less than 10 dBm/500 MHz according to ② the peak PSD regulation. In addition, when there are multiple pulses during a 1-ms period, the average power, which is the accumulated effect of these pulses, must be less than −14.3 dBm/500 MHz according to ① the average PSD regulation.

The reason for having two criteria is because the average power changes depending on the number of pulses. For example, if there is only one pulse with a bandwidth of 500 MHz per 1 ms, as shown in Figure 1a, then the peak power will have a rather large value of 42.7 dBm/500 MHz under ① the average PSD regulation. This means that although the average output is small, the peak power is quite large, and thus it can interfere with other wireless devices for a short period of time. Therefore, according to local regulation ②, the peak power must be limited to 10 dBm or less even if there is one pulse. Accordingly, the case where both local regulations are satisfied is when the peak power is 10 dBm and the number of pulses is 1862 for a UWB pulse with a bandwidth of 500 MHz, as shown in Figure 1b. In this case, the average power becomes −14.3 dBm/500 MHz, and thus both local regulations ① and ② are satisfied. Regarding spectral emissions, the IEEE 802.15.4z standard does not specify a mandatory adjacent channel power rate (ACPR) or spectral mask requirements, unlike narrowband communication standards. Therefore, our link-budget optimization focuses solely on complying with the average and peak power constraints defined by worldwide UWB regulations.

**Figure 2:**
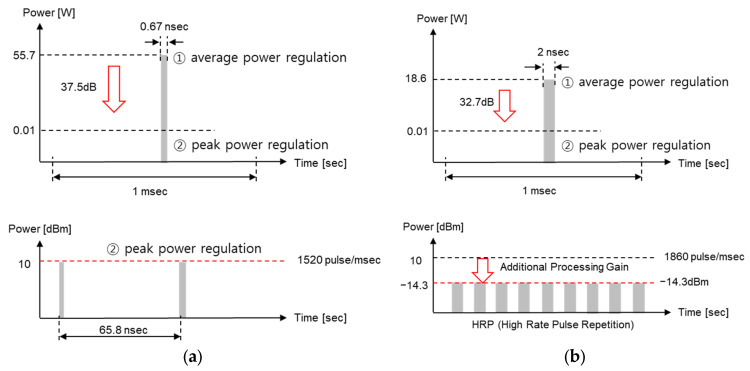
Pulse characteristics of UWB radar and HRP UWB communication (**a**) in the case of UWB radar and (**b**) in the case of HRP UWB communication.

Now, based on this local regulation for the output power, let us find out the number of pulses per 1 ms, peak power, and average power when a UWB device is used for communication or radar. First, since UWB radar measures the distance by using the time difference between the transmitted pulse and the pulse backscattered from the target, the pulse repetition frequency (PRF) is determined by the maximum measurement distance. For example, Novelda’s UWB x4 module, a representative UWB radar module, uses a pulse with a bandwidth of 1.5 GHz (pulse width of approximately 0.67 ns) and waits for the pulse for 65.8 ns after transmitting the pulse [30]. When this time is converted into a distance, it is 9.87 m. Since the actual operating range of Novelda’s UWB radar is only 1–2 m, it is possible to use more pulses, but in this case, the average power exceeds −14.3 dBm/MHz and thus cannot be used; that is, the pulse number and peak power of the UWB radar are determined by local regulations ① and ②, as shown in Figure 2a.

Next, the currently commercialized IEEE 802.15.4z-based UWB communication method is HRP UWB with a relatively high PRF [7]. In Figure 1, when the peak power is 10 dBm, the number of pulses with a bandwidth of 500 MHz possible for 1 ms is 1862, and using more pulses than this number is called HRP UWB. As shown in Figure 2b, when the number of pulses increases, the peak power becomes smaller than the value of local regulation ②. Instead, by using a large number of pulses, additional processing gain can be obtained through the spread spectrum. However, since the number of pulses cannot be increased indefinitely, it must be limited so as to not exceed the average power suggested in local regulation ①. In other words, in order to increase the processing gain, the more pulses the better, but the number of pulses cannot exceed the local regulation for the average power. Since UWB communication must maintain −14.3 dBm according to the average power under local regulation ①, HRP UWB communication becomes a system whose performance is limited only by the average power regardless of the peak power.

## 3. Link Analysis of HRP UWB Communication

### 3.1. Packet Structure of HRP UWB

**Figure 3:**
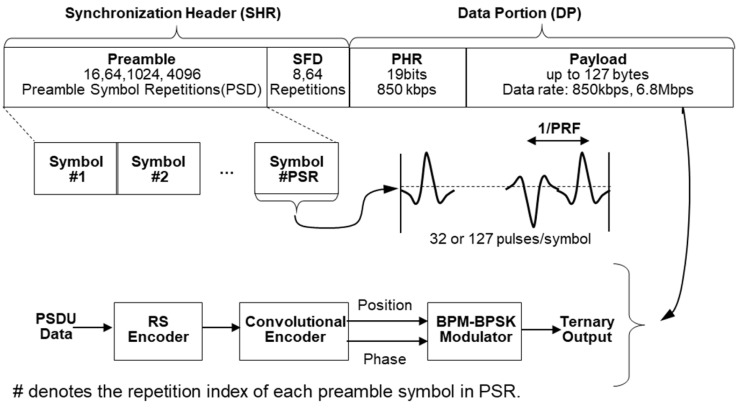
Typical packet structure of IEEE 802.15.4z HRP UWB.

In the previous section, we examined how HRP UWB communication is a system whose performance is limited only by the average power. In this section, we will examine how this principle is implemented in the actual IEEE 802.15.4z HRP UWB standard. Figure 3 shows the packet structure presented in the IEEE 802.15.4z HRP UWB standard [5,7]. As shown in Figure 3, the packet structure of IEEE 802.15.4z HRP UWB is divided into a synchronization header (SHR) used to set the reference time for synchronization and distance measurement and a data portion (DP) for transmitting data, and both use separate modulation methods. The IEEE 802.15.4z HRP UWB standard also includes a scrambled time sequence (STS) for encrypted distance measurement, but since STS uses the same modulation as the SHR, Figure 3 shows a packet without an STS.

First, the SHR is divided into a preamble and start-of-frame delimiter (SFD). The preamble is transmitted by repeating the same symbol, and its length is determined by the number of preamble symbol repetitions (PSRs). One preamble symbol is defined by 31 or 127 sub-symbols, and each sub-symbol has a value of (−1, 0, 1). Here, ‘1’ means a UWB pulse with positive (+) polarity, ‘−1’ means a UWB pulse with negative (−) polarity, and ‘0’ means no pulse. These pulses have the time interval of the inverse of the PRF. The basic PRF (BPRF) mode of the IEEE 802.15.4z standard uses a PRF of 64 MHz. Next, the SFD consists of 8 or 64 symbols and indicates the end of the preamble and the start of the DP. The preamble can obtain processing gain by repeatedly sending a known pulse without any special modulation. In HRP UWB, this is defined as the number of packet acquisition chunks (PACs). In addition to the processing gain through the PACs, the preamble uses the leading edge detection (LED) algorithm to increase the ranging accuracy; that is, if the number of PSRs is 1024, and the PAC count is 32, then one channel impulse response (CIR) is generated by collecting the preambles of 32 PACs, and the LED algorithm is used to increase the ranging accuracy by using 32 (=1024/32) of these CIRs [1].

**Figure 4:**
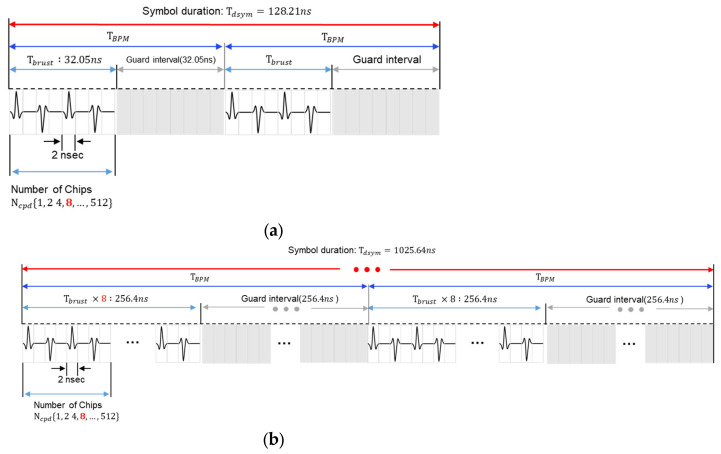
HRP UWB PHY BPM-BPSK modulation structure and symbol duration: (**a**) data rate of 6.8 Mbps and (**b**) data rate of 850 kbps.

Next, the DP consists of a physical header (PHR) and a payload. The PHR includes information about the length and speed of payload data and uses different channel coding and modulation schemes from the SHR. Reed–Solomon (RS) coding and convolutional coding are used for channel coding, and burst position modulation–binary phase shift keying (BPM-BPSK) modulation is used as a modulation method. The DP supports multiple data rates, including 850 kbps and 6.8 Mbps. The difference between the 850 kbps and 6.8 Mbps modes in the IEEE 802.15.4z standard is achieved by adjusting the symbol duration and the internal burst structure while maintaining the same transmission bandwidth. For example, the 850 kbps mode adopts a much longer symbol duration with more burst positions and chips per symbol. In contrast, the 6.8 Mbps mode uses a shorter symbol duration with fewer burst positions and chips, allowing faster data transmission at the expense of the link margin. The symbol structures for the 850 kbps and 6.8 Mbps data rates are shown in Figure 4.

This is because unlike the SHR, pulses are not sent repeatedly in the data section, and thus there is no processing gain; rather, coding gain is obtained through channel coding to balance the link between the preamble and payload. In HRP UWB communication, the ranging limit is determined by the smaller link margin between the SHR and DP links, making it necessary to adjust the parameters to appropriately balance the two.

### 3.2. Communication and Ranging Principles of HRP UWB

**Figure 5:**
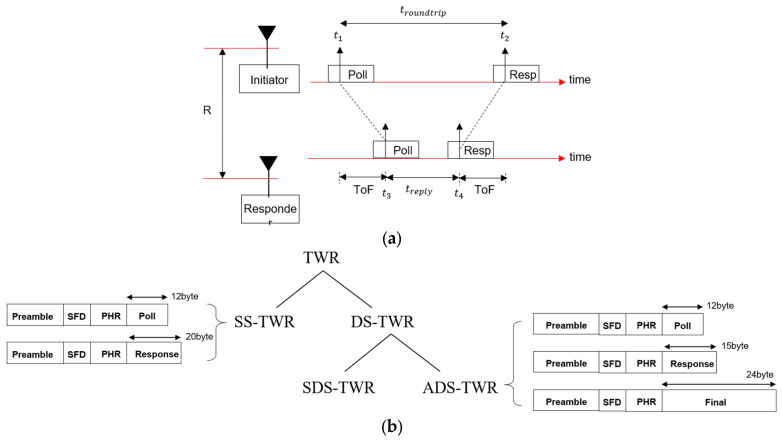
Two-way ranging: (**a**) principle of UWB two-way ranging and (**b**) classification of HRP UWB two-way ranging and related packets.

Section 3.1 explains the structure and modulation method of the physical layer packet of the IEEE 802.15.4z HRP UWB standard. This section examines the principle of how HRP UWB technology measures distance based on wireless communication. Since HRP UWB is basically a wireless communication method, packets are exchanged to send and receive data. Among the packets, the preamble performs the role of synchronization for data demodulation, and the payload among DPs is used for data transmission. The principle of ranging between UWB devices using this packet exchange is as shown in Figure 5a. Figure 5a is a method of measuring distance by exchanging only two packets, which is called single-sided two-way ranging (SS-TWR). Among the two UWB devices, the device that sends the packet first is called the initiator, and the other is called the responder. The initiator transmits a Poll packet and records the time $t_{1}$ when the transmission of the Poll packet begins, while $t_{1}$ is the moment when the preamble ends, and this time record is called a timestamp. The UWB responder device that received the Poll message sends a Response packet after the time $t_{reply}$. Now, the UWB initiator records the time at which the Response packet was received as a timestamp $t_{2}$. Now, the distance between the two UWB devices is 1/2 of the time of propagation (ToF), which is given by Equation (1):

(1) $$R=c\times \frac{t_{2}-t_{1}-t_{reply}}{2},$$

where c means the speed of light. In this way, ranging using two packets is possible through wireless communication, and this is called two-way ranging (TWR).

Meanwhile, the types of TWR that measure distance in the UWB can be divided into SS-TWR, which exchanges packets twice (Poll-Response), and double-sided-TWR (DS-TWR), which exchanges packets three times (Poll-Response-Final), as shown in Figure 5b. DS-TWR is further classified into symmetrical DS-TWR (SDS-TWR) and asymmetrical DS-TWR (ADS-TWR). Among these, ADS-TWR, which can freely use the length of the packet, is mainly used for ranging. ADS-TWR is used when high distance accuracy is required because it can minimize the influence of clock errors between the transmitter and receiver. In ADS-TWR, the length of the packet is the shortest for the Poll packet and the longest for the Final packet, as shown in Figure 5b. Regardless of the TWR method used, in order to measure the distance between UWB devices with TWR, two packets must be successfully exchanged for SDS-TWR, and three packets must be successfully exchanged for DS-TWR. To this end, both the preamble and the payload of the packets must be successfully received.

### 3.3. Link Analysis of Data Portion in TWR Procedure

In order for the TWR process of HRP UWB explained in Section 3.2 to be successful, packets related to ranging must first be successfully exchanged. To this end, there must be no preamble errors and no DP data errors. In other words, the link budget of the preamble and the link budget of the DP must be considered simultaneously.

First, let us look at the DP link. The link performance of the DP is determined by whether the packet reception is successful or not and is usually given as the packet error rate (PER). In this study, we assume a PER of 1% or more as the link performance criterion. This value is a value commonly discussed in the current next-generation UWB standard IEEE 802.15.4ab [8]. The PER performance of BPM-BPSK used in UWB communication is generally given as shown in Equation (2) [31]:

(2) $$PER=NQ\left(\sqrt{\gamma \frac{E_{b}}{N_{0}}}\right)$$

where N is the number of bits per packet, γ is the coding gain, $E_{b}$ is the bit energy, and $N_{0}$ is the power density spectrum of noise. As shown in Figure 5b, the ‘Poll’, ‘Response’, and ‘Final’ packets are required for ADS-TWR, and the longest packet is the ‘Final’ packet with a size of 24 bytes. Thus, this packet should be used as the criterion for PER evaluation; that is, since *N* is 192 bits, $E_{b}/N_{0}$ of the DP corresponds to 9.0 dB, assuming 1% PER. Next, the IEEE 802.15.4z HRP UWB standard uses convolutional channel coding and Reed–Solomon channel coding, and thus it has a channel coding gain of about 5.6 dB [14]. Therefore, the DP of the IEEE802.15.4z HRP UWB standard should have an $E_{b}/N_{0}$ value of about 3.4 dB [23]. In addition, since the IEEE802.15.4z HRP UWB standard spreads one bit over multiple chips, the signal-to-noise ratio (SNR) is given by Equation (3):

(3) $$SNR=\frac{E_{b}}{N_{0}}\frac{R_{b}}{BW}=\frac{E_{b}}{N_{0}}\left(\frac{R_{b}}{R_{c}}\right)$$

where BW is the bandwidth, $R_{b}$ is the bit rate, and $R_{c}$ is the chip rate. Based on the IEEE 802.15.4z HRP UWB standard, when $R_{b}$ is 850 kbps, the SNR is calculated to be about −24.3 dB, and when $R_{b}$ is 6.8 Mbps, the SNR is calculated to be −15.3 dB. In other words, if the data rate is as low as 850 kbps, then there is a 9 dB link margin, and thus a wider communication range can be achieved than in the case of 6.8 Mbps.

**Figure 6:**
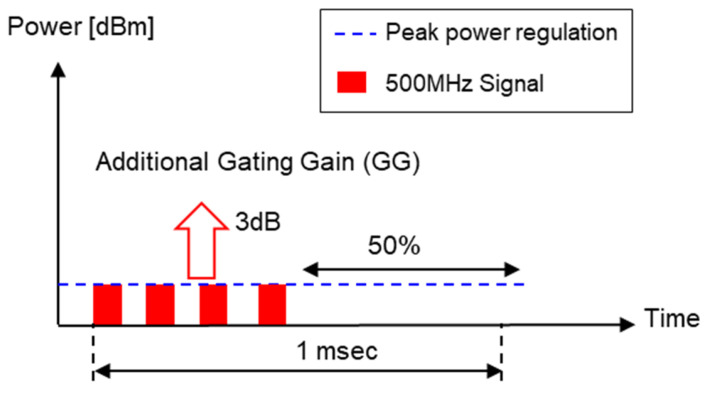
Example of increased output power via gating gain.

Now that we know the SNR value, let us analyze the DP link based on the transmitter parameters of the IEEE 802.14z HRP UWB standard and the power regulation in Table 2. The transmitted power is given by Equation (4):

(4) $$P_{TX,DP}\left[dBm\right]={PSD}_{TX}+BW+GG$$

where $P_{TX,Preamble}$ is the transmitted power of the preamble in dBm, ${PSD}_{TX}$ is the average power regulation of −41.3 dBm/MHz, BW is the bandwidth in dBMHz, and GG is the gating gain. The gating gain is given by Equation (5):

(5) $$GG\left[dB\right]=10{log}_{10}\left(\frac{1[ms]}{T_{p}}\right)$$

where $T_{p}$ corresponds to the time length of the packet. In other words, if the packet length is less than 1 ms, it indicates that the output can be increased by that amount according to the local regulation. Figure 6 shows a case where the output power is doubled by GG when $T_{p}$ is 0.5 ms. Typically, UWB IC manufacturers such as Qorvo and NXP provide a function that can control power through the gating gain if the packet length is shorter than 1 ms [23]. Finally, the receiver sensitivity is given by Equation (6):

(6) $$S_{DP}\left[dBm\right]=-174+BW+NF+{SNR}_{min}$$

where ${SNR}_{min}$ is the minimum SNR, which is given by Equation (3), and NF is the receiver’s noise figure. Finally, the received power is calculated based on the Friis transmission formula, which is a path loss model in free space.

### 3.4. Link Analysis of Preamble in TWR Procedure

**Figure 7:**
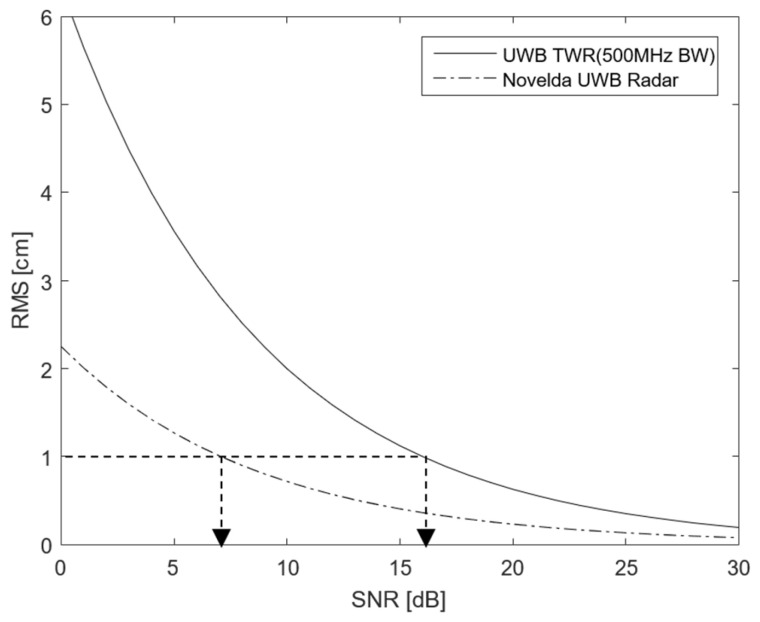
Cramer–Rao bound (CRB) for IEEE 802.15.4z HRP UWB ranging. The arrow indicates the SNR value at which the theoretical lower bound (CRB) reaches 1 cm, highlighting the required link quality for high-accuracy ranging.

In Section 3.3, we analyzed the link characteristics in the DP of HRP UWB. In this section, we analyze the ranging accuracy when data are successfully exchanged. In UWB communication, the ranging accuracy is related to the preamble, and the root mean square (RMS) value of the ranging accuracy of the preamble is given by the Cramer–Rao bound (CRB). In general, the RMS of the ranging accuracy is given as a function of the SNR and effective bandwidth as shown in Equation (7) [31,32]:

(7) $$RMS\left(d\right)\geq \sqrt{CRB}=\frac{c}{2\sqrt{2}\pi {BW}_{eff}\sqrt{SNR}}$$

where c is the speed of light, SNR is the signal-to-noise ratio of the preamble, and ${BW}_{eff}$ is the effective bandwidth, which corresponds to 500 MHz in the case of the IEEE 802.15.4z HRP UWB standard [9]. Based on Equation (7), the ranging accuracy is expressed as a function of the SNR and effective bandwidth as shown in Figure 7.

If the ranging accuracy is 1 cm, then the SNR of the UWB preamble requires 16 dB according to Equation (7). In other words, unlike the SNR value required by the DP in the UWB packet, the preamble requires a 12.6 dB higher SNR value. To this end, the SNR value is increased by repeatedly transmitting the same symbol in the preamble. At this time, the processing gain is given by the number of symbol repetitions as shown in Equation (8):

(8) $$PG\left[dB\right]=10{log}_{10}\left(N_{p}\right)$$

where $N_{p}$ means the number of symbols used for ranging in the preamble. The number of symbols to be used for the processing gain of Equation (8) among the number of symbols in the preamble is defined by the PSRs and PACs. Now, the transmitted power of the preamble of the HRP UWB is given as shown in Equation (9) because the processing gain is added, unlike Equation (4):

(9) $$P_{TX,Preamble}\left[dBm\right]={PSD}_{TX}+BW+GG+PG$$

where $P_{TX,Preamble}$ is the transmitted power of the preamble in dBm, ${PSD}_{TX}$ is the average power regulation of −41.3 dBm/MHz, BW is the bandwidth in dBMHz, and GG and PG are the gating gain and the processing gain, respectively. Finally, the received power is based on the Friis transmission formula, which is a path loss model in free space.

**Figure 8:**
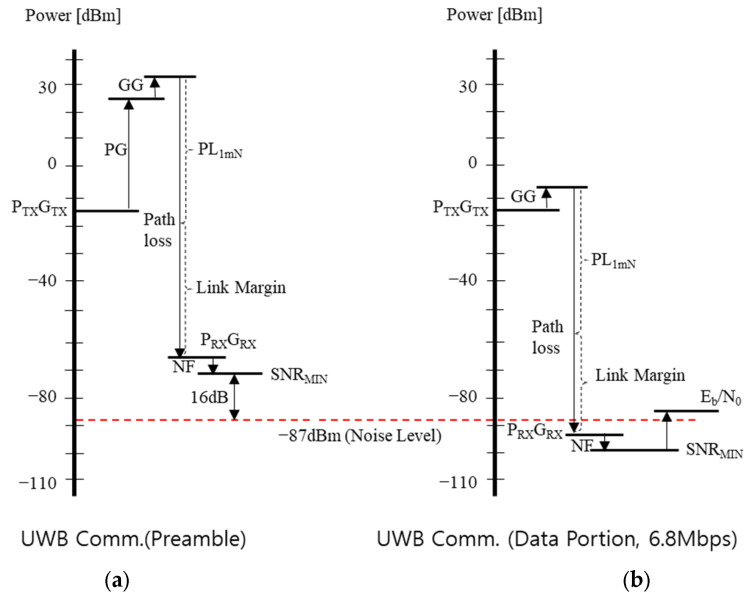
Link budget of IEEE 802.15.4z HRP UWB (**a**) in the case of the preamble link and (**b**) in the case of a 6.8 Mbps data portion.

Now, using Equations (3)–(9), the link budget for each preamble and the DP of IEEE 802.15.4z UWB communication are calculated as shown in Table 3. In Table 3, CH9, which is currently available worldwide, was used as the UWB frequency. Among the results in Table 3, the preamble link and the DP link with a data transmission rate $R_{b}$ of 6.8 Mbps are represented graphically as shown in Figure 8. As can be seen in Table 3, the link performance of the preamble was better than that of the DP. In other words, when the distance increases, the preamble may be received, but the data link may be broken. This has been experimentally presented in related papers [33,34]. In addition, it can be seen that in the DP, sending data at a low speed of 850 kbps rather than a data rate of 6.8 Mbps can improve the performance by about 9 dB. This is consistent with the results provided by the chip manufacturer [23].

**Figure 9:**
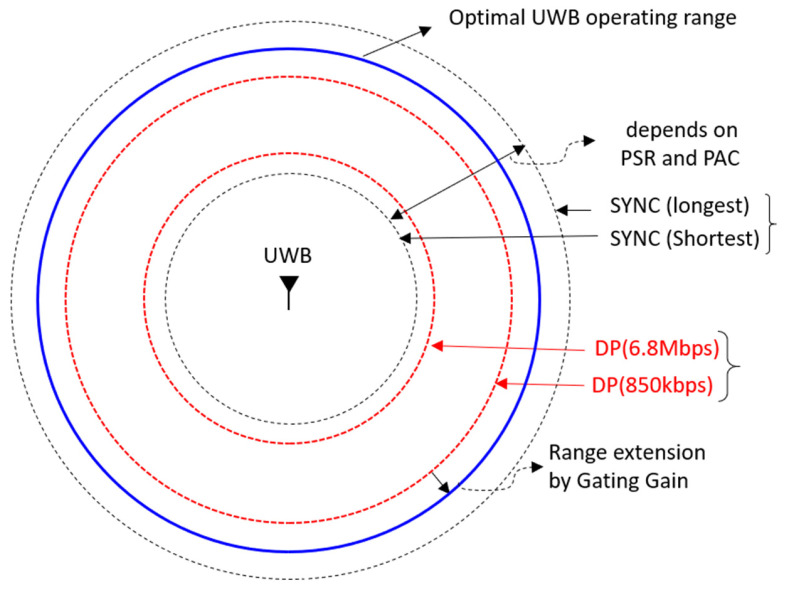
Summary of ranging coverage extension concepts for IEEE 802.15.4z HRP UWB.

Our analysis results are summarized in Figure 9. The ranging coverage of UWB communication at a data rate of 850 kbps is more than twice as large as that for a data rate of 6.8 Mbps. The link performance of the preamble, which is the basis for synchronization and ranging, varies depending on the length of the preamble. In the IEEE 802.15.4z HRP UWB standard, the length of the preamble varies from a minimum of 16 to a maximum of 4096, and thus a longer preamble has larger ranging coverage than a shorter preamble. However, since the message must be successfully received for two-way ranging, the characteristics of the preamble need to be appropriately adjusted to the link of the data portion. After the link balances of the DP section and the SYNC section are matched, it is possible to additionally increase the transmission power using the gating gain if the packet length is less than 1 ms. This has been confirmed to be consistent with the results of many experimental papers. The results of this study can help to adjust the physical layer parameters when installing actual HRP UWB products to increase the ranging coverage.

## 4. Experimental Results and Discussion

In Section 3, the link performance required in the preamble and DP was theoretically analyzed. In this section, we verify the theoretical results through experiments using an actual HRP UWB module. In the experiments, we verify the performance changes according to the changes in PHY parameters such as the PSR, PAC, and data rate (850 kbps versus 6.8 Mbps) and the possibility of longer ranging. In particular, we verified the following results examined in the theoretical analysis through this experiment. First, since the link margin of the preamble is theoretically higher than that of the DP, errors may occur in the DP of the packet at a long distance, but the preamble-based synchronization signal will still be able to be received. Second, low-speed data (850 kbps) will show about 9 dB of performance gain compared with high-speed data (6.8 Mbps), as analyzed in the theory. Third, the processing gain will be increased according to the increase in the PAC size and PSR length, which will improve the distance measurement accuracy and stability. To verify these three points, the experiment was conducted as follows.

### 4.1. Experiments on Link Performances at a Fixed Distance

**Figure 10:**
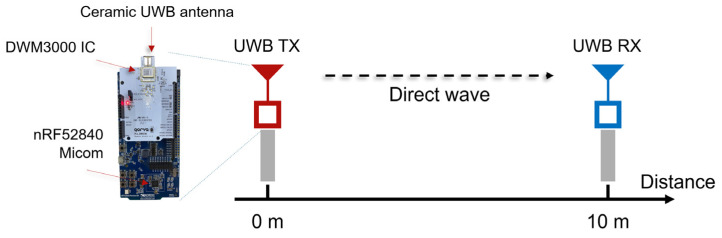
Link experiment set-up at 10 m using DW3000 UWB module.

Figure 10 shows the hardware used in the experiment and the communication link’s experimental environment at a fixed distance of 10 m. The Qorvo DW3000 HRP UWB IC was controlled by an NRF52840-DK microcontroller board, and the PHY parameters, such as the PSRs, PACs, and data rate, could be flexibly set through the embedded programming of the microcontroller. The output of the transmitter was adjusted in 1 dB units to reproduce various link conditions. In the first experiment, the SHR reception rate (HRR) and the packet reception rate (PRR), including the SHR and DP, were measured while changing the parameters and transmitter output at a fixed distance of 10 m to verify the link budget difference between the preamble and DP. Through this, the difference in the reception of the preamble and DP of the packet was quantitatively compared. The experiment used the SP0 packet format of IEEE 802.15.4z, and the RF parameters were set as specified in Table 4. In this experiment, the PSR count was fixed to 128, and the PAC count was fixed to 8. Qorvo’s DW3000 UWB IC provides a function to adjust the transmit power to compensate for the difference between the RF output of the chip and the actual RF output and antenna loss [23]. For example, it supports a maximum output of −41.3 dBm/MHz while complying with the spectrum emission regulations and is adjustable from 0 dB up to −30 dB. In this experiment, packets were transmitted while adjusting the TX power gain of the transmitter in 1 dB units. In order to analyze the reception status of the HRP UWB IC and the success or failure of packet reception, register values such as Receiver PHY Header Detect (RXPHD) and Receiver PHY Header Error (RXPHE) of the system status register (SYS_STATUS) were monitored, and 200 packets were transmitted for each experimental condition. Through this experiment, we could find out whether the packet was received and the error rate of the DP and compare and analyze the PER performance between the preamble and the DP.

**Figure 11:**
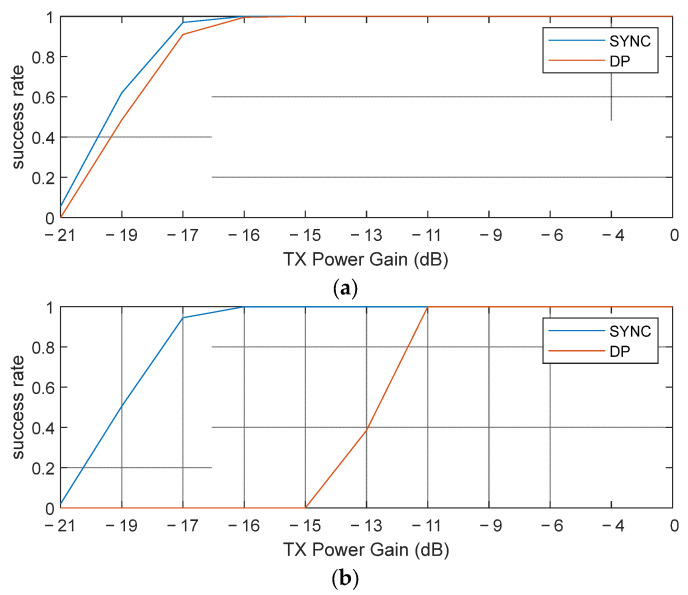
Experiment results of HRP UWB at 10 m using DW3000 UWB module: (**a**) low-speed (850 kbps) data transmission and (**b**) high-speed (6.8 Mbps) data transmission.

Figure 11 shows the packet reception characteristics as a function of the transmission power through the coarse and fine gain adjustments in the DW3000 IC. The experimental results show that a reception success rate of about 80% or higher was achieved from −17 dB at 850 kbps, while the same reception success rate was achieved from about −11 dB at 6.8 Mbps. This means that the low-speed (850 kbps) data transmission provided about 6 dB of link gain compared with the high-speed (6.8 Mbps) data transmission. The reason why a gain of 6 dB was observed, which was slightly smaller than the theoretically suggested 9 dB, was probably due to the interaction between the synchronization performance in the SYNC (preamble) and the reception performance in the DP, which was not considered in the link analysis; that is, in the case of 6.8 Mbps, stable reception was possible in the SYNC, but due to an insufficient link margin in the DP, there was an overall link performance difference of about −6 dB, whereas in the case of 850 kbps, the reception performances of the SYNC and DP sections were similarly close, showing that the insufficient link margin of the SYNC also affected the DP performance. Through this, the link budget difference between the preamble and DP was clearly revealed in the experiment, and the link difference according to the change in data transmission speed could be quantitatively confirmed.

### 4.2. Maximum Ranging Experiment According to PSR and PAC Size

**Figure 12:**
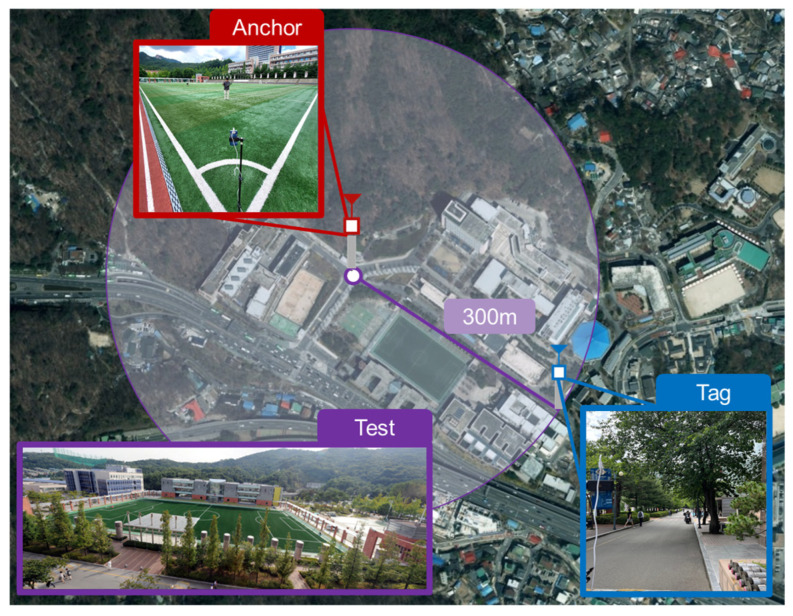
Long ranging experiment set-up.

The second experiment was conducted to identify the maximum communication distance and stable ranging measurement conditions in an actual sports field environment. The measurement environment was the athletic field of Kookmin University in Seoul, Republic of Korea, as shown in Figure 12. The RF environment at the athletic field was verified to be free of active RF sources, and no significant interference was expected, as WiFi 6E operation is restricted to indoor use under local regulations in Korea. The maximum ranging distance satisfying a packet reception success rate of 90% was measured by changing parameters such as the PSR and PAC counts, and the distance values at that time were organized as median values. Through this process, it was possible to confirm how the processing gain according to the PSR and PAC counts affected the actual long-distance ranging performance. The experimental conditions are as shown in Table 4, and the same SP0 packet format as that in the first experiment was used, but the ranging measurement was performed with the DS-TWR method. The preamble used low-speed data transmission (850 kbps) to be suitable for long-distance ranging. Through these settings, it was possible to confirm how much the processing gain in the preamble contributed to the actual maximum ranging measurement. The coverage of ranging satisfying a success rate of 90% or more was compared and analyzed by changing the PSR and PAC values step by step.

**Figure 13:**
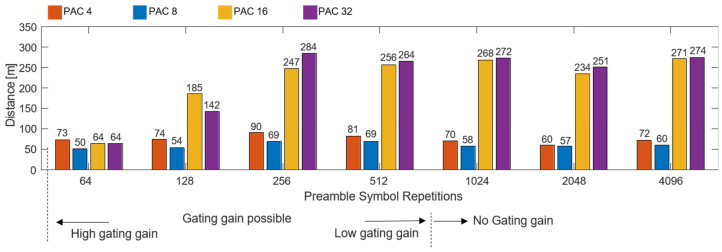
Maximum measurable distance according to variations in PSR and PAC sizes based on a 90% success rate.

Figure 13 shows the results for the maximum ranging distance according to the PAC size and PSR length. As can be seen in the figure, the interaction between the PACs and PSRs had a significant impact on the ranging performance. The ranging performance comparison according to the change in PAC size showed excellent performance in the order of 32, 16, 4, and 8 PACs, and in particular, the lowest overall performance was shown when the PAC size was 8. It was confirmed that the maximum ranging performance improved by 2–3 times just by changing the PAC size from 8 to 16. This confirms that the processing gain secured by the increase in PAC size greatly contributes to longer ranging coverage. In addition, Figure 13 shows that the link performance of the SYNC and DP was balanced at 256 PSRs, as analyzed in Figure 10, and there was no significant change even if the PSR count was increased beyond that. Therefore, this shows that the operating range could be further expanded through the gating gain after fixing the PSR count to 256. In an experiment where the output was increased after fixing the PSR count to 256, it was confirmed that stable ranging measurement was possible at a distance of more than 300 m.

As shown in Figure 13, the ranging coverage increased as the PSR count increased in the order of [64, 128, 256, 512, 1024, 4096]. However, the PSR effect was found to be interrelated with the PAC size. Specifically, when the PAC size was set to four or eight, the distance measurement performance tended to remain below 100 m overall. On the other hand, when the PAC size was set to 16 or 32, the distance measurement capability was significantly improved as the PSR count increased. In particular, the performance improvement effect was remarkable when the PSR count increased in the order of [64, 128, 256], and the best distance measurement performance was observed overall when the PAC size was set to 32. However, the performance improvement due to the increase from 16 to 32 was relatively limited compared with when the PAC size was increased from 8 to 16.

Through this experiment, it was found that increasing the PAC and PSR counts significantly improved the link margin in the SHR section, leading to a significant improvement in the maximum distance measurement. However, if the link margin of the DP was relatively insufficient, even if the gain was secured in the SYNC section, the success rate was rapidly reduced beyond a certain distance, showing a limitation. In addition, as the PSR count increased, the processing gain in the SYNC section increased, but the overall packet length increased, which may hinder resource utilization in terms of gating gain utilization and simultaneous access performance of multiple tags. This means that the link margins of the SYNC and DR sections are complementary, and the optimization of both areas must be performed simultaneously during system design to simultaneously maximize the actual long-distance measurement stability and performance. In summary, this experiment verified that the long-distance measurement performance and stable distance measurement capability can be significantly improved by adjusting the PAC size and PSR length of the preamble while keeping the DP speed at a low speed of 850 kbps. This shows that PAC and PSR parameter optimization is necessary when designing a UWB-based long ranging system.

### 4.3. Maximum Ranging Experiment in Indoor Environments

To evaluate the practical ranging performance of the proposed physical layer parameter adjustments under real-world indoor conditions, additional experiments were conducted in the hallway of the engineering building of Kookmin University in Seoul, Korea. The measurement environment consisted of a straight hallway (line-of-sight (LOS) dominant, with significant multipath effects) with a length of approximately 90 m. Multipath propagation in the straight hallway caused increased signal spreading, slightly affecting the maximum achievable ranging distance. However, in the NLOS corner area, preamble detection failures occurred much more frequently, leading to a substantial reduction in the maximum measurable distance.

Three different physical layer parameter configurations were tested to comprehensively assess the ranging performance. For each case, experiments were conducted at both the 850 kbps and 6.8 Mbps data rates to investigate the impact of the transmission speed on ranging performance. The details of the tested parameter settings are summarized in Table 5.

**Figure 14:**
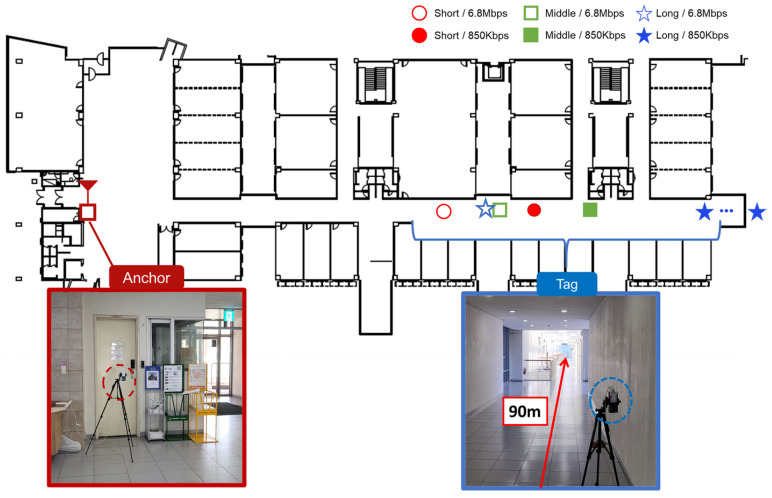
Maximum ranging distances mapped onto the hallway layout of the engineering building.

Figure 14 presents the maximum ranging distances achieved under each experimental condition overlaid on the building’s floor plan. Different marker shapes and colors represent the physical layer settings; red circles indicate the short case, green squares represent the baseline (middle) case, and blue stars correspond to the long case. Solid markers denote the results at 850 kbps, while hollow markers represent the results at 6.8 Mbps. This visualization highlights the influence of the physical layer parameters and data rate on the achievable ranging distances in real-world indoor environments.

The experimental results are summarized in Table 6, which shows the maximum achievable distances under each configuration based on a packet reception success rate of 90%. At a data rate of 6.8 Mbps, both the long and middle cases exhibited a rapid decrease in ranging performance, with distance measurement failures occurring near 65 m. In contrast, the short case showed an earlier failure at approximately 62.3 m, which was slightly shorter than its 850 kbps maximum distance (68.8 m), likely due to the link budget limitations of the DP. When operating at 850 kbps, the ranging performance improved significantly, as the link budget of the DP was no longer the dominant limiting factor. Instead, the PSR became the main contributor to the ranging capability. As a result, the maximum achievable distances followed the order of short, middle, and long cases, directly reflecting the impact of the PSR length on preamble detection performance under multipath conditions. In particular, the long case successfully achieved distance measurements up to the end of the hallway (90 m) with high reliability, and the results suggest that even longer distances would be possible if the physical space allowed them. These observations confirm that the selection of both the DP parameters and the PSR critically impacted the maximum achievable ranging performance. Furthermore, it was demonstrated that reliable extended UWB ranging is achievable even in real-world indoor environments with significant multipath effects, such as a university campus hallway.

## 5. Conclusions

In this study, we analyzed the link performance of the IEEE 802.5.4z HRP UWB standard, which has been commercialized in various fields recently, from the perspective of the link budget and proposed a method for configuring the optimal HRP UWB parameters for long-distance ranging up to hundreds of meters. Since UWB technology allows for various wireless link designs as long as they satisfy the technical criteria, we confirmed that ranging up to hundreds of meters is possible if designed optimally. The results analyzed in this study were tested with an actual commercial UWB module using a non-directional antenna. Therefore, if an actual directional antenna is used, then the possibility of long-distance ranging of hundreds of meters or more will increase even more.

From a system design perspective, it is important to recognize that, despite the wide bandwidth (~500 MHz) characteristic of UWB systems, significant link performance asymmetry exists between the synchronization header and the data portion due to their different modulation schemes. While conventional assumptions often treat UWB links as uniformly robust across the packet, our analysis and experimental results demonstrate that the weaker link of the two—typically the DP—ultimately constrained the maximum achievable ranging distance. Therefore, future UWB system designs and standard developments should explicitly account for this inherent link margin disparity. By addressing and compensating for the weaker link through adaptive modulation, coding, or dynamic parameter tuning strategies, further improvements in ranging performance, reliability, and coverage can be realized without violating regulatory constraints. In future UWB system designs, explicitly addressing the link margin imbalance between the SHR and DP sections will be key to maximizing performance and deployment scalability. We expect that UWB technology can be used in various applications that require long-distance ranging in the future.

## Figures and Tables

**Table 1:** Key performance comparisons of previous UWB research results.

Vendor	Chip or Module	Ranging Method	Frequency (GHz)	Max Range (m)	Reference
Qorvo	DWM1000	TWR, TDoA	3.5–6.5	~50	[19,20,21,22]
Qorvo	DW3000	TWR, TDoA	6.5, 8	~50	[16,17,23]
Qorvo	DW3120	TWR, TDoA, PDoA	6.5, 8	~50	[18,24]
NXP	SR150	TWR, TDoA, PDoA	6.24–8.24	~50	[25]
NXP	SR040	TWR, TDoA	6.24–8.24	~50	[26]
Spark	SR1010	TWR	3.1–6	~100	[27]
Spark	SR1020	TWR	6–9.3	~100	[27]
Qorvo	DW3000	TWR	6.5	~300	This Work

**Table 2:** Local regulation for UWB worldwide.

	Local Regulation
Frequency	3.1–10.6 GHz ^1^
Average power	−41.3 dBm/MHz@1 ms ^①^
Peak power	0 dBm/50 MHz ^②^
Bandwidth	More than 450 MHz (1 MHz RBW, 10 dB bandwidth)

^1^ Frequency ranges vary slightly from country to country. ^①^ Regulation for average Power Spectral Density (PSD) over 1 ms, typically specified as −41.3 dBm/MHz or −14.3 dBm/500 MHz. ^②^ Regulation for peak power, requiring pulses not to exceed 10 dBm/500 MHz even if only a single pulse is transmitted.

**Table 3:** Link budget calculation of IEEE 802.15.4z HRP UWB.

	Preamble	Data Portion (850 kbps)	Data Portion (6.8 Mbps)
Code length	127	-	-
No. of symbols	256	-	-
PAC size	32	-	-
Preamble length [us]	268.6	-	-
PRF [MHz]	64	62.4	62.4
Coding gain [dB]	-	5.6	5.6
Coded $E_{b}/N_{0}$ [dB]	-	3.5	3.5
Payload [bits]	-	160	160
Packet length	-	306.4	100.9
PSD [dBm/MHz]	−41.3	−41.3	−41.3
Bandwidth [MHz]	499.2	499.2	499.2
Frequency [GHz]	7.98	7.98	7.98
NF [dB]	6	6	6
PG [dB]	36.1	-	-
GG [dB]	0	0	0
$P_{TX}$ [dBm]	−14.3	−14.3	−14.3
${SNR}_{min}$ [dB]	10.0	−21.6	−12.5
S [dB]	−106.7	−105.5	−96.5
Path Loss [dB]	92.6	91.1	82.1
${PL}_{1m}$ [dB]	50.5	50.5	50.5
Link margin	42.2	40.7	31.7

**Table 4:** Configuration PHY settings in DW 3000 UWB module.

PHY Setting	Values
RF channel	CH5
PRF	64 MHz
PSR	32, 64, **128**, 256, 512, 1024, 2048, 4096
PAC	4, **8**, 16, 32
DP	850 kbps, 6.8 Mbps

**Table 5:** Experimental parameter configurations for ranging performance evaluation.

Case	PSR	PAC	DP
Short	64	4	850 kbps, 6.8 Mbps
Middle	256	4	850 kbps, 6.8 Mbps
Long	4096	32	850 kbps, 6.8 Mbps

**Table 6:** Maximum achievable ranging distance.

Case	6.8 Mbps	850 Kbps
Short	62.3 m	68.8 m
Middle	66 m	71.77 m
Long	65.5 m	Above 90 m

## Data Availability

Data are contained within the article.

## Abbreviations

The following abbreviations are used in this manuscript:

UWB	Ultra-wideband
HRP	High-rate pulse repetition frequency
WPAN	Wireless personal area network
PSD	Power spectral density
PRF	Pulse repetition frequency
SHR	Synchronization header
DP	Data portion
STS	Scrambled time sequence
SFD	Start-of-frame delimiter
PSR	Preamble symbol repetitions
PAC	Packet acquisition chunks
LED	Leading edge detection
CIR	Channel impulse response
PHR	Physical header
RS	Reed–Solomon
BPM-BPSK	Burst position modulation–binary phase shift keying
SS-TWR	Single-sided two-way ranging
DS-TWR	Double-sided two-way ranging
SDS-TWR	Symmetrical DS-TWR
ADS-TWR	Asymmetrical DS-TWR
PER	Packet error rate
SNR	Signal-to-noise ratio
RMS	Root mean square
CRB	Cramer–Rao bound
ACPR	Adjacent channel power ratio
